# Development of PCRSeqTyping—a novel molecular assay for typing of *Streptococcus pneumoniae*

**DOI:** 10.1186/s41479-017-0032-3

**Published:** 2017-05-25

**Authors:** Geetha Nagaraj, Feroze Ganaie, Vandana Govindan, Kadahalli Lingegowda Ravikumar

**Affiliations:** Central Research Laboratory, KIMS Hospital and Research Centre, KR Road, VV Purum, Bangalore, Karnataka 560 004 India

**Keywords:** Molecular serotyping, PCRSeqTyping, *Streptococcus pneumoniae*, cpsB sequencing

## Abstract

**Background:**

Precise serotyping of pneumococci is essential for vaccine development, to better understand the pathogenicity and trends of drug resistance. Currently used conventional and molecular methods of serotyping are expensive and time-consuming, with limited coverage of serotypes. An accurate and rapid serotyping method with complete coverage of serotypes is an urgent necessity. This study describes the development and application of a novel technology that addresses this need.

**Methods:**

Polymerase chain reaction (PCR) was performed, targeting 1061 bp cpsB region, and the amplicon was subjected to sequencing. The sequence data was analyzed using the National Centre for Biotechnology Information database. For homologous strains, a second round of PCR, sequencing, and data analysis was performed targeting 10 group-specific genes located in the capsular polysaccharide region. Ninety-one pneumococcal reference strains were analyzed with PCRSeqTyping and compared with Quellung reaction using Pneumotest Kit (SSI, Denmark).

**Results:**

A 100% correlation of PCRSeqTyping results was observed with Pneumotest results. Fifty-nine reference strains were uniquely identified in the first step of PCRSeqTyping. The remaining 32 homologous strains out of 91 were also uniquely identified in the second step.

**Conclusion:**

This study describes a PCRSeqTyping assay that is accurate and rapid, with high reproducibility. This assay is amenable for clinical testing and does not require culturing of the samples. It is a significant improvement over other methods because it covers all pneumococcal serotypes, and it has the potential for use in diagnostic laboratories and surveillance studies.

## Background

*Streptococcus pneumoniae*, found in the upper respiratory tract of healthy children and adults, causes a range of infections including meningitis, septicemia, pneumonia, sinusitis, and otitis media. Children < 2 years of age and adults aged ≥65 years of age are particularly susceptible [1]. According to the Morbidity and Mortality Weekly Report, April 26 2013 [2], an estimated 14.5 million cases of serious pneumococcal disease (including pneumonia, meningitis, and sepsis) occur each year in children aged <5 years worldwide, which has resulted in approximately 500,000 deaths, mostly in low- and middle-income developing countries.

The high morbidity and mortality caused by pneumococci are not clearly understood. The pathogenicity of pneumococci has been linked to various virulence factors such as capsule, cell wall and its component polysaccharides, pneumolysin, PspA, complement factor H-binding component, autolysin, neuraminidase, peptide permeases, hydrogen peroxide, and IgA1 protease [3–5]. Capsular polysaccharide (CPS) is the primary virulence factor, and is also used to categorize, *S. pneumoniae*into more than 90 different serotypes [6–8]. Capsule is important for the survival of bacteria at infection site as it provides resistance to phagocytosis [9].

Pneumococcal CPS is generally synthesized by the Wzx/Wzy-dependent pathway, except for types 3 and 37, which are produced by the synthase pathway [10, 11]. Most genes required for synthesis of capsule are within the capsule polysaccharide synthesis (*cps*) operon, which ranges from 10 kb (serotype 3) to 30 kb (serotype 38). *Cps* operon is flanked by *dexB* in 5′ end and *aliA* at 3′ end. Neither of these participates in capsule synthesis. The 5′-end of the CPS loci starts with regulatory and processing genes *wzg, wzh, wzd,* and *wze* (also known as *cpsABCD*), which are conserved with high sequence identity in all serotypes, followed by the central region consisting of serotype specific genes [12, 13].

Pneumococcal serotyping is necessary for epidemiological and vaccine impact studies. It also aids in understanding the pathogenicity of the organism and closely monitors for the emergence of non-vaccine strains, replacement serotypes, and new serovars [14, 15]. Widespread use of pneumococcal vaccines has led to replacement with serotypes that are not included in the vaccines. Continuous monitoring of serotypes is therefore essential for epidemiological surveillance and long-term vaccine impact studies [16–20].

Several phenotypic and genotypic methods are currently used to identify pneumococcal group and type. The phenotypic serotyping methods of capsular swelling reaction, latex agglutination and coagglutination tests are costly, require skilled personnel, and cannot detect all serotypes. Genotypic typing methods that assess genome variation include sequential multiplex polymerase chain reaction (PCR), sequential real-time PCR, restriction fragment length polymorphism (RFLP), microarray, sequetyping, and matrix-assisted lazer desorption ionization-time of flight (MALDI-TOF) analysis. In addition to general applicability and a high discriminatory power, these genotypic assays are economical, detect pneumococci directly from the clinical specimen, and detect emerging serovars, replacement strains, and vaccine escape recombinants [21]. However, many of these methods are multistep, intricate, and do not discriminate all serotypes [22–26].

It is crucial to develop a robust, simple method with complete serotype coverage for serotype detection and pneumococcal serogroup/serotype surveillance [27]. Herein, the authors describe an innovative serotyping approach that relies on sequencing of assembly genes located in the capsular operon to identify all pneumococcal serotypes.

## Methods

### Reference strains

There were 91 reference serotype strains of *S. pneumoniae* obtained from Staten Serum Institute, Copenhagen, Denmark (Table 1).

**Table 1:** PCRseqtyping results for 91 SSI strains

Sl. NO	Serogroup	Serotype	NCBI ACCESSION NO	PCRSeqTyping results	
				Step I	Step 2
1	1	1	CR931632	1	
2	2	2	CR931632	2/41A	2
3	3	3	CR931634	3	
4	4	4	CR931635	4	
5	5	5	CR931637	5	
6	6	6A	CR931638	6A	
7		6B	CR931639	6B	
8		6C	EF538714	6C	
9	7	7 F	CR931643	7 F	
10		7A	CR931640	7A	
11		7B	CR931641	7B/40	7B
12		7C	CR931642	7C	
13	8	8	CR931644	8	
14	9	9A	CR931645	9A/9 V	9A
15		9 L	CR931646	9 L	
16		9 N	CR931647	9 N	
17		9 V	CR931648	9A/9 V	9 V
18	10	10 F	CR931652	10 F/10C	10 F
19		10A	CR931649	10A	
20		10B	CR931650	10B	
21		10C	CR931651	10 F/10C	10C
22	11	11 F	CR931657	11 F	
23		11A	CR931653	11A/11D/18 F	11A
24		11B	CR931654	11B	
25		11C	CR931655	11C	
26		11D	CR931656	11A/11D/18 F	11D
27	12	12 F	CR931660	12 F/44	12 F
28		12A	CR931658	12A	
29		12B	CR931659	12B	
30	13	13	CR931661	13/20	13
31	14	14	CR931662	14	
32	15	15 F	CR931666	15 F	
33		15A	CR931663	15A	
34		15B	CR931664	15B	
35		15C	CR931665	15C	
36	16	16 F	CR931668	16 F	
37		16A	CR931667	16A	
38	17	17 F	CR931670	17 F	
39		17A	CR931669	17A/34	17A
40	18	18 F	CR931674	11A/11D/18 F	18 F
41		18A	CR931671	18A	
42		18B	CR931672	18B	
43		18C	CR931673	18C	
44	19	19 F	CR931678	19 F	
45		19A	CR931675	19A	
46		19B	CR931676	19B	
47		19C	CR931677	19C	
48	20	20	CR931679	13/20	20
49	21	21	CR931680	21	
50	22	22 F	CR931682	22 F/22A	22 F
51		22A	CR931681	22 F/22A	22A
52	23	23 F	CR931685	23 F	
53		23A	CR931683	23A	
54		23B	CR931684	23B	
55	24	24 F	CR931688	24 F	
56		24A	CR931686	24A	
57		24B	CR931687	24B	
58	25	25 F	CR931690	25 F/25A	25 F
59		25A	CR931689	25 F/25A	25A
60	27	27	CR931691	27	
61	28	28 F	CR931693	28 F	
62		28A	CR931692	28A	
63	29	29	CR931694	29	
64	31	31	CR931695	31	
65	32	32 F	CR931697	32 F/32A	32 F
66		32A	CR931696	32 F/32A	32A
67	33	33 F	CR931702	33 F/33A/35A	33 F
68		33A	CR931698	33A	
69		33B	CR931699	33B	
70		33C	CR931700	33C	
71		33D	CR931701	33D	
72	34	34	CR931703	17A/34	34
73	35	35 F	CR931707	35 F/47 F	35 F
74		35A	CR931704	33 F/33A/35A	35A
75		35B	CR931705	35B/35C	35B
76		35C	CR931706	35B/35C	35C
77	36	36	CR931708	36	
78	37	37	CR931709	37	
79	38	38	CR931710	38	
80	39	39	CR931711	39	
81	40	40	CR931712	7B/40	40
82	41	41 F	CR931714	41 F	
83		41A	CR931713	2/41A	41A
84	42	42	CR931715	42	
85	43	43	CR931716	43	
86	44	44	CR931717	44	
87	45	45	CR931718	45	
88	46	46	CR931719	46	
89	47	47 F	CR931721	35 F/47 F	47 F
90		47A	CR931720	47A	
91	48	48	CR931722	48	

### Clinical isolates

There were 28 clinical isolates of *S. pneumoniae* selected from isolates submitted to Central Research Laboratory, KIMS Hospital, Bangalore (Table 2). They were isolated from blood (*n* = 23), cerebrospinal fluid (CSF) (*n* =3) and pleural fluid (*n* = 2).

**Table 2:** Serotype distribution of the clinical isolates of *Streptococcus pneumoniae* from Central Research Laboratory, KIMS Hospital, Bangalore, India

Sl.No	Sample ID	SEX	AGE YRS	SOURCE	PCRSeq Typing data	Quellung data	Homologous(H) & Non-homologous (NH)
1	PIDOPS-01	M	5	Blood	6B	6B	NH
2	PIDOPS-02	F	2y5m	Blood	14	14	NH
3	PIDOPS-03	M	5	Pleural fluid	7 F	7 F	NH
4	PIDOPS-04	M	6 m	Blood	20	20	H- HG5
5	PIDOPS-05	M	5	Blood	14	14	NH
6	PIDOPS-07	M	1y6m	CSF	15B	15B	NH
7	PIDOPS-08	M	2	Blood	19 F	19 F	NH
8	PIDOPS-09	M	4y3 m	Blood	19 F	19 F	NH
9	PIDOPS-10	M	2y2 m	Blood	6B	6A	NH
10	PIDOPS-11	M	5	Blood	6B	6B	NH
11	PIDOPS-14	M	3	Blood	1	1	NH
12	PIDOPS-17	M	1y6m	Blood	19 F	19 F	NH
13	PIDOPS-18	M	4	Blood	1	1	NH
14	PIDOPS-19	M	1y6m	Blood	1	1	NH
15	PIDOPS-20	M	3	Blood	1	1	NH
16	PIDOPS-22	F	9 m	Blood	6A	6A	NH
17	PIDOPS-23	F	3y8m	Blood	11A	11A	H-HG1
18	PIDOPS-24	M	5	Blood	8	8	NH
19	PIDOPS-25	F	4y6m	CSF	5	5	NH
20	PIDOPS-28	M	9 m	Blood	1	1	NH
21	PIDOPS-30	F	3y3 m	Blood	15B	15B	NH
22	PIDOPS-31	M	3 m	Blood	19A	19A	NH
23	PIDOPS-32	M	2y6m	Pleural fluid	19A	19A	NH
24	PIDOPS-33	M	4y2 m	Blood	7B	7B	H-HG2
25	PIDOPS-42	M	2 m	CSF	6B	6B	NH
26	PIDOPS-45	F	6 m	Blood	7 F	7 F	NH
27	PIDOPS-46	F	10 m	Blood	19A	19A	NH
28	PIDOPS-50	M	5y	Blood	3	3	NH

### Media and culture conditions

Strains were stored in skim milk, tryptone, glucose, and glycerol (STGG) media at −80 °C. They were cultured on 5% sheep blood agar (Chromogen, Hyderabad) for 18–24 hrs at37°C with 5% CO_2_. The isolates were characterized as *S. pneumoniae* by colony morphology, alpha hemolysis, bile solubility, and optochin susceptibility.

### Serotyping

Quellung reaction was performed using Pneumotest kit and type-specific antisera (SSI, Denmark), as recommended by the manufacturer.

### PCRSeqTyping

PCRSeqTyping assay was performed in two steps. Step I involved PCR amplification and sequencing of the *cpsB* gene from genomic DNA. There were 91 serotypes that were divided into non-homologous group (Group I, 59 serotypes) and homologous group (Group II, 32 serotypes) based on the *cpsB* sequence data. The homologous group was further subdivided into 10 subgroups based on the sequence homology. The second step involved PCR and sequencing of each homology group by using specific primers in order to identify the unique serotypes.

### Nucleic acid extraction

Genomic DNA was extracted from bacterial strains using QIAamp DNA mini kit (Qiagen, Germany), as per the manufacturer’s protocol.

### PCR amplification

PCR reaction was performed using the primers designed by Leung et al. [26] with modifications. Primers used in the study were *cps1-FP (*5′-GCAATGCCAGACAGTAACCTCTAT-3)′, *cps2-RP (*5′-CCTGCCTGCAAGTCTTGATT-3′) and cps-2538-RP (5′-CTTTACCAACCTTTGTAATCCAT-3′). The reaction mixture was modified to contain 50–100 ng of genomic DNA, 0.75 units XT-5 polymerase (3 U/μl, Merck, which is a mixture of thermo stable enzymes Taq DNA polymerase and proof-reading [PR] polymerase), 1X XT5A-Assay buffer, 1 μl deoxynucleoside triphosphates (dNTPs, 2.5 mM each [Fermentas, United States]), 1 μl forward primer (100 ng/μl), 1 μl of reverse primer mix (100 ng/μl). The final reaction volume was made up to 25 μl with DNase/RNase-free distilled water (Gibco, United States). Thermal cycling was performed in GeneAmp PCR system 9700 (Applied Biosystems, United States) under the following conditions: 94 °C for 5 min, followed by 35 amplification cycles of 94 °C for 30 s, 50 °C for 30 s, and 72 °C for 1 min and final extension at 72 °C for 5 min. The PCR products were separated by electrophoresis on 1.2% agarose gel for 45 min at 80 V in 1X Tris-acetate EDTA buffer. Ethidium bromide-stained DNA products were visualized under ultraviolet (UV) illumination and size of the DNA products was determined by using a 1–kb DNA molecular size marker (Fermentas).

### Sequencing and data analysis

PCR products were purified using QIA quick PCR purification kit (Qiagen, Germany) following manufacturer’s protocol. Purified PCR products were subjected to sequencing, employing the Big Dye Sequence Terminator kit V3.1 (Applied Biosystems) and analyzed on ABI 3730 XL Genetic Analyzer (Applied Biosystems). Sequencing was performed in one direction using forward primer (cps1), 5′-GCA ATG CCA GAC AGT AAC CTC TAT-3′ and Long Seq Module (ABI). DNA sequences that were obtained were analyzed for sequence similarity using GenBank database (http://www.ncbi.nlm.nih.gov/blast) and then assigned to serotype [26]. Serotype of the *cpsB* nucleotide sequence was determined from GenBank with the highest BLAST bit score of > 99% sequence identity with the query ‘amplicon nucleotide sequence’.

### Homology group assignment and PCRSeqTyping

#### Homology groups

**Fig. 1:**
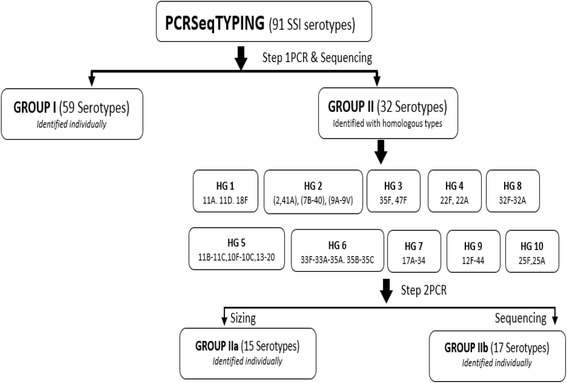
Homology group assignment for 91 pneumococcal serotypes

Amplifiable serotypes that shared identical interceding sequences (e.g. sequences for serotypes 2 and 41A, 7B, and 40) were grouped into 10 different groups based on their homology by *in silico* analysis of *cpsB* region. Individual primer sets were designed for each subgroup. Sequetyping data obtained in Step I was used to assign the homologous strains into subgroups (Fig. 1). Serotypes were considered homologous when the highest bit score was shared between two or more serotypes (i.e. the same amount of nucleotide variation between query and database sequences), and then assigned to one of the 10 groups (Table 3).

**Table 3:** Primers used in PCRSeqTyping assay

	Primers		Sequence (5′-3′)	Product size (in bp)	Serotype
GROUP I	FP1	CPS-1FP	GCAATGCCAGACAGTAACCTCTAT	1061	
	RP1	CPS-2RP	CCTGCCTGCAAGTCTTGATT		
	RP2	2538-RP	CTTTACCAACCTTTGTAATCCAT	1109	
GROUP II					
HG1	HG1-FP	FP1	TGTCCAATGAAGAGCAAGACTTGAC	1109	11A
	HG1-RP	RP1	AAGTATATCCCTCCACAAACCCATC	435	11D
				316	18 F
HG2	HG2-FP	FP1	TGTCCAATGAAGAGCAAGACTTGAC	1628	2
	HG2-RP	RP5	ATATCACTTTTTTACGGTAATGTCTA	1820	41A
				1185	7B
				1820	40
				1819	9 V
				1502	9A
HG3	HG3-FP	FP1	TGTCCAATGAAGAGCAAGACTTGAC	1797	35 F
	HG3-RP	RP7	CACCTTTATTTTCACTATCTGCATC	1479	47 F
HG4	HG4-FP	FP8	ACTAGGAAGCTAGCCGTAGGTTGC	366	22 F
	HG4-RP	RP8	TCTCACCTTTAGTGCTTGAACCT	No Amplification	22A
HG5	HG5-FP	FP9	CCATGGGATGCTTTCTGTGTGGA	1061	10 F
	HG5-RP	RP9	TATATCACTTTTTTACGGTAATGTCTA	1004	10C
				1416	11B
				2958	11C
				1395	13
				1395	20
HG6	HG6-FP	FP1	TGTCCAATGAAGAGCAAGACTTGAC	929	33 F
	HG6-RP	RP4	AGCACCTAGCACCTGTTTAGAT	929	33A
				924	35A
				927	35B
				925	35C
HG7	HG7-FP	FP3	CAGAGTTCGTCTTACTTGGCAGCT	737	34
	HG7-RP	RP3	GAATCTTGCAAGCTATTAATGATCG	737	17A
HG8	HG8-FP	FP6	AGCAACTAGCCAAGTTAGCCAGAGT	643	32 F
	HG8-RP	RP6	ACTGTGCTTCCATCTGGGACATCATG	648	32A
HG9	HG9-FP	FP1	TGTCCAATGAAGAGCAAGACTTGAC	970	12 F
	HG9-RP	RP2	CAGAAAAAGTAGCCTTATTTCTTAAGA	996	44
HG10	HG10-FP	FP10	ATGAAGCTATTCAAAGTTTGTTAGC	656	25 F
	HG10-RP	RP10	TGAATCCTCTAATCCTTGCATGA	656	25A

For homologous strains, a second round of PCR was performed using group specific primers as specified in Table 3. PCR products were subjected to sequencing reaction. The nucleotide sequence data was used to assign the serotype.

## Results

### PCRSeqTyping results for reference strains

The 91 pneumococcal serotype reference strains (sourced from SSI) were tested with PCRSeqTyping protocol*.* All 91 strains were amplified using the modified method. In Step I of amplification and sequencing, 59 strains of the non-homologous group (Group I) were correctly assigned to their respective serotype. There were 32 strains (Group II) identified along with their homologous type. The homologous types were correctly assigned to their respective type in Step II by performing a second round of amplification using group specific primers and sequencing. Quellung reaction performed using Pneumotest kit (SSI), in parallel with PCRSeqTyping, showed 100% concordant results (Table 1).

The results were further evaluated by blinded testing of PCRSeqtyping. Samples were evaluated randomly by assigning codes. Quellung reaction data showed no discrepancies between serotypes assigned by Quellung and PCRSeqTyping for all reference strains.

### PCRSeqTyping results for clinical isolates

Twenty eight pneumococcal isolates tested in the study were from children <5 years with invasive pneumococcal disease. The predominant serotypes were 1, 6B, 19A, 19 F, 14 and 7 F (Table 2). PCRSeqTyping results and serotyping results by Quellung reaction were in concordance, without any discrepancies. Among 28 isolates, 25 isolates were assigned to their serotype with the first step of PCRSeqTyping. Three isolates belonging to the homologous group were subsequently identified with the second step of PCRSeqTyping.

## Discussion

There is a renewed interest in pneumococcal capsular typing techniques, as a result of an increased complexity in the management of pneumococcal disease and the widespread use of pneumococcal vaccines [8]. The ability to differentiate pneumococcal strains efficiently is essential to track the emerging serovars, and for epidemiological investigations. The limitations of the Quellung serotyping method, many DNA-based typing protocols, PCR, restriction fragment length polymorphisms, hybridization assays, microarrays and sequencing for *S. pneumoniae* are well known. Different PCR strategies, namely multiplex PCR, sequential PCR, serotype-specific PCR, and real time multiplex PCR [25, 28–36] targeting serotype-specific regions of *cps* could detect only 22 serotypes uniquely, and 48 serotypes along with their homologous types [37, 38]. Despite the fact these methods cover imited serotypes, PCR is a widely used technique, which avoids the use of serological reagents and requires specific expertise to conduct.

Methods using multiple restriction enzymes and long *cps* fragments [39, 40] for PCR make the amplification difficult and inconsistent. Another protocol based on sequencing of regulatory region of *cps* [30, 31] shows poor resolution with cross reactivity of serotypes. An approach targeting serotype-specific glycosyl transferase genes [6] was only tested for serogroup 6 and serotype 19 F. The cross reactivity of serotypes, along with the requirement for a higher number of primers, and poor resolution limits their wide usage.

With the characterization of the *cps* locus of 92 serotypes [13], Leung et al. [26] developed sequetyping protocol using single primer pair, which binds in all pneumococcal serotypes. Recently, several research groups [27, 41–43] have published their results using sequetyping assay. Limitations of the sequetyping protocol were as follows: (i) only 84 serotypes out of 92 were predicted to be amplified by *in silico* analysis; (ii) cross-reacting serotypes (30/84) belonging to homologous groups could not be uniquely identified; and (iii) considering the central 732 bp region of the *cpsB* amplicon which could be sequenced, only 46 of 54 serotypes could be sequetyped.

In the first step of this study’s modified approach, successful amplification of all 91 serotypes was achieved with the addition of a new reverse primer to amplify 25A, 25 F and 38 serotypes specifically. Additionally, XT-5 polymerase used in the PCR amplification reactions contains *Taq* DNA polymerase and *Pfu* enzyme. This enzyme blend utilizes the powerful 5′-3′ polymerase activity of Taq DNA polymerase and the 3′-5′ exonuclease-mediated proof-reading activity of PR polymerase, resulting in high fidelity PCR products [44]. PCR annealing temperature of 50 °C and extension time of 1 min were found to be optimal for amplification of *cpsB* gene of all 91 strains.

The serotypes were grouped into homologous (32) and non-homologous (59) based on *cpsB* sequence. Non-homologous types were identified uniquely. The 32 homologous strains were further subdivided into 10 groups (HG 1–10) based on their sequence similarity. Homology group-specific primers were designed and evaluated for their ability to differentiate between strains. HG primers were designed to be able to assign the serotype accurately with second step of PCR and sequencing.

The limitation of using 732 bp region of *cpsB* amplicon in sequetyping assay, resulting in prediction of 46 of 54 serotypes, was overcome with the use of Long Seq module. Approximately 1.0 kb quality reads in a single sequencing reaction were obtained with modification. This resulted in providing good quality reads up to the end of the PCR template, identifying cross-reacting serotypes (15B/15C, 7 F/7A, 18B/18C, 9 L/9 N, 15B/C, 17 F/33C, 18B/C, 7A/F, 12A/46, 6C/6D) which have a single SNP in the *cpsB* region.

A 100% concordance of serotype results of PCRSeqTyping and Quellung testing was seen for the 28 clinical isolates. Moving forward, the study will be extended for serotyping a larger number of clinical isolates and clinical samples. The limitation of the protocol will be in quantification and serotype identification in multiple carriage; however, studies are underway to address these issues. For multiple carriage, the PCR amplicon obtained in the first step will be subcloned into T/A cloning vector and the individual clones will be sequenced for assigning the specific serotype. As the corresponding *cpsB* gene sequence of the recently discovered serotypes 6E, 6 F, 6G, 6H, 11E, 20A, 20B and 23B1 [45–47] were unavailable at the time of the study design, they will be included in future studies.

In the study’s center, the typing cost with Pneumotest Kit (SSI, Denmark) was US$35/isolate, while PCRSeqTyping cost was US$10 for Group I (non-homologous strains) and US$15 for Group II (homologous strains). With the easy availability of outsourced sequencing services, the accurate and reliable PCRSeqTyping test can be adopted in a regular microbiology laboratory, even without the sequencing facility.

This modified typing method has several advantages over other reported methods. It involves techniques with a workflow that many microbiology laboratories can easily implement. The high throughput PCRSeqTyping method features good discriminatory power, reproducibility, and portability, making it suitable for epidemiological studies. The assay has the flexibility of incorporating additional primers for the characterization of emerging serotypes. An added advantage of this method is that raw data from experiments can be reanalyzed upon the addition of new entries to the serotyping database.

## Conclusion

PCRSeqTyping assay is a cost-effective alternative to currently available phenotypic and molecular typing methods. The method is simple to perform, robust, and economical. It can identify all 91 serotypes specifically and uniquely.

## Abbreviations

Cps: Capsular polysaccharide
DNA: DeoxyRibo Nucleic Acid
EDTA: Ethylenediaminetetraacetic acid
MALDI-TOF: Matrix Assisted Laser Desorption Ionization - Time of Flight
PCR: Polymerase chain reaction
RFLP: Restriction fragment length polymorphism
SSI: Staten Serum Institute
STG: Serotype/group
STGG: Skim milk, tryptone, glucose, and glycerol
